# Vitamin D deficiency among patients with COVID-19: case series and recent literature review

**DOI:** 10.1186/s41182-020-00277-w

**Published:** 2020-12-20

**Authors:** Rizaldy Taslim Pinzon, Angela, Andryawan Wahyu Pradana

**Affiliations:** 1 Duta Wacana Christian University School of Medicine, Dr. Wahidin Sudirohusodo street number 5-25, Yogyakarta, 55224 Indonesia; 2 Bethesda Hospital Yogyakarta, Jendral Sudirman street number 70, Yogyakarta, 55224 Indonesia

**Keywords:** COVID-19, Coronavirus, Vitamin D, Vitamin D deficiency, Low vitamin D

## Abstract

**Background:**

The world is now challenging the pandemic of COVID-19 infection. This is the third and most extensive pandemic. Previous studies showed the plausibility of vitamin D prophylaxis and therapy for COVID-19, particularly in settings where hypovitaminosis D is frequent. Recent study from Indonesian showed that the prevalence of vitamin D deficiency was 23.0%. The examination of vitamin D status is not a routine in the Indonesian clinical setting.

**Methods:**

This study is a case series from confirmed cases of COVID-19 in Bethesda Hospital Yogyakarta Indonesia. The data of clinical symptoms, signs and laboratory examinations were obtained from the electronic medical records. The vitamin D status was measured by Enzyme-Linked Fluorescent Assay (ELFA) method. We searched PubMed and Google Scholar for studies that included terms for Vitamin D and COVID-19.

**Results:**

The data were obtained from 10 participants consisting of 50% male and 50% female. The mean age was 49.6 years. The prevalence of vitamin D deficiency in this study was 90% (vitamin D levels < 20 ng/mL) and 10% of insufficiency (vitamin D levels < 30 ng/mL). Patients in this study had various symptoms such as fatigue (60%), fever (50%), dry cough (40%), non-specific headache (10%), and diarrhea (10%); have no symptoms (20%); and also had the various chronic diseases as comorbidity such as hypertension (40%), diabetes (10%), COPD (10%), and post stroke (10%).

**Conclusions:**

All of the COVID-19 patients in this study had hypovitaminosis D. The prevalence of vitamin D deficiency in this case series is 90% and only 1 patient (10%) had vitamin D insufficiency. There are many health benefits of vitamin D and very few adverse effects. Randomized controlled trials need to determine and evaluate this recommendation in preventing or treating COVID-19. Clinicians should continue to treat people with vitamin D deficiency especially in managing COVID-19 patients.

## Background

The world is now challenging the pandemic of coronavirus (CoV) infections, the disease called COVID-19. A new CoV infection epidemic began in Wuhan, China, in late 2019 [1]. This is the third and most extensive pandemic after the first severe acute respiratory syndrome (SARS)-CoV, which started in China in 2002 [2]. The second epidemic from coronavirus was the Middle East respiratory syndrome (MERS)-CoV in the Middle East, first reported in 2012 [3].

The data from the Indonesian task force for COVID-19 on 16 June 2020 showed that there were 40,400 confirmed cases, 22,466 active suspected cases, and 2231 death (5.5%). The mortality rate is the highest among Southeast Asian countries [4].

Indonesia is a tropical country with abundant sun exposure, as it lies within the equatorial zone. A previous study showed that the prevalence of 25-hydroxyvitamin D [25(OH)D] deficiency among Indonesian elderly women in institutionalized care is approximately 35.1% [5]. A recent study from Indonesia showed that the prevalence of vitamin D deficiency was 23.0% [6].

Vitamin D has been proven to enhance the expression of antioxidation-related genes, modulate adaptive immunity, and improve cellular immunity [7, 8]. Vitamin D has immuno-modulatory properties that include downregulation of proinflammatory cytokines, and has been shown to attenuate lipopolysaccharide-induced acute lung injury in mice by blocking effects on the angiopoietin (Ang)-2-Tie-2 signaling pathway and on the renin-angiotensin pathway [8, 9]. The protective effect of vitamin D against COVID-19 is related to the suppression of the cytokine response and reduced severity/risk for ARDS. The evidence from a meta-analysis that regular oral vitamin D_2_/D_3_ intake (in doses up to 2000 IU/d without additional bolus) is safe and protective against acute respiratory tract infection, especially in subjects with vitamin D deficiency [10].

There has been no proven therapy for COVID-19 yet. Previous studies have shown the plausibility of vitamin D prophylaxis and therapy for COVID-19, particularly in settings where hypovitaminosis D is frequent. The examination of vitamin D status is not a routine in the Indonesian clinical setting. The aim of our study was to report a case series of vitamin D status in patients with confirmed COVID-19 and review recent literature on the role of vitamin D in COVID-19.

## Methodology

### Research design

This original article was case series from confirmed COVID-19 cases in Bethesda Hospital Yogyakarta, Indonesia. The aim of this study is to explain that the majority of COVID-19 patients have low vitamin D levels, which may contribute to decreased immunity.

We also do recent literature review by searched PubMed and Google Scholar for studies that included terms for vitamin D and COVID-19. We found no trials of vitamin D in COVID-19 that have reported results. We did find several studies that are registered but have not yet been reported. None seemed to be masked comparisons to placebo.

### Research subjects

The data were obtained consecutively from the COVID-19 confirmed real-time PCR patients that admitted to Bethesda Hospital Yogyakarta, Indonesia. The data of clinical symptoms, clinical signs, and laboratory examinations were obtained from the electronic medical records and analyzed descriptively.

### Laboratory analysis

The vitamin D status was measured by a standardized laboratory method. The vitamin D examination was performed using VIDAS 25(OH)D (bioMerleux, Marcy l’Etolie, France) for vitamin D_2_ and D_3_ with an enzyme-linked fluorescence assay. The high precision of VIDAS 25(OH)D (bioMerleux, Marcy l’Etolie, France) shows by CV < 16% from 8 to 20 ng/mL and CV < 5% from 20 to 126 ng/mL. The lowest amount of 25(OH)D can be quantitatively determined with the stated accuracy of CV < 20%. The city of the company (bioMerleux, Marcy l’Etolie, France) is in France [11].

The measurement of 25-hydroxyvitamin D [25(OH)D] serum was performed by enzyme immunoassays for the quantitative measurement of total serum of 25-hydroxyvitamin D [25(OH)D] levels. The 3 groups of vitamin D status include deficiency status (25(OH)D levels < 10 ng/mL), insufficiency status (25(OH)D levels 10–29 ng/mL), and sufficiency status (25(OH)D levels 30–100 ng/mL).

### Statistical analysis

This case series is a descriptive study that explains vitamin D deficiency among patients with COVID-19. To determine the deficiency status in COVID-19 patients, we used the normal values from the laboratory results of vitamin D level examination as an indicator of vitamin D status. We also analyzed vitamin D levels based on demographics and laboratory examinations data using the Fisher’s exact test. Data analysis was carried out by SPSS Statistics version 23.

## Results

### Case presentation

Herein, we report that there were 10 participants with COVID-19 consisting of 50% males (5 patients) and 50% females (5 patients). All patients had either positive serological or real-time PCR tests for COVID-19. The average age of the participants was 49.6 years old. All patients involved in this study had body mass index (BMI) of less than 25. Symptoms felt by patients vary, and some are asymptomatic. About 60% of patients had symptoms of fatigue, 50% with fever, 40% with dry cough, 10% with headache, 10% with non-specific headache, 10% with diarrhea, and 20% had no symptoms but had close contact with COVID-19 patients. All patients had mild to moderate severity. The patients’ comorbidities also vary, 40% have a history of hypertension, 10% patients with diabetes, 10% patients with COPD, and 10% patients with post stroke.

Based on the laboratory tests, 90% (9 patients) had vitamin D deficiency status (vitamin D levels < 20 ng/mL), 3 out of 10 patients had vitamin < 8.1 ng/mL, and 10% (1 patient) had vitamin D insufficiency status (vitamin D levels 20–29 ng/mL) and there were no patients with normal or adequate vitamin D levels. The results of other blood tests can be seen in Tables 1 and 2.

**Table 1:** Demographics, clinical characteristics on admission, and laboratory results of 10 patients cases with COVID-19 infection

	Patient 1	Patient 2	Patient 3	Patient 4	Patient 5	Patient 6	Patient 7	Patient 8	Patient 9	Patient 10
**Demographics**										
**Age (years)**	49	51	17	40	65	73	14	54	69	64
**Mean of age = 49.6 years**										
**Gender**	Female	Male	Male	Male	Female	Male	Female	Female	Female	Male
**Clinical findings on admission**										
**Symptom**	Fatigue(close contact)	Fever, diarrhea	No symptoms (close contact)	Fever, fatigue, dry cough	Fever, fatigue, headache	Fever, fatigue, dry cough,	No symptoms, (close contact)	Fatigue, non-specific headache	Fatigue, dry cough	Fever, dry cough
**Comorbidity**	-	Diabetes	-	-	Hypertension	Hypertension, COPD	-	-	Hypertension	Post stroke, hypertension
**SARS-CoV IgG**	Positive	Positive	Positive	Positive	Positive	Positive	Positive	Positive	Positive	Negative
**SARS-CoV IgM**	Positive	Positive	Positive	Positive	Positive	Positive	Positive	Positive	Positive	Positive
**Laboratory results**										
**Vitamin D levels (ng/mL)**	< 8.1	10.6	20.5	11.9	11.6	12.4	8.3	10.1	< 8.1	< 8.1
**Vitamin D status**	**Deficiency**	**Deficiency**	**Insufficiency**	**Deficiency**	**Deficiency**	**Deficiency**	**Deficiency**	**Deficiency**	**Deficiency**	**Deficiency**
**Hemoglobin (g/dL)**	12.4	16	17.6	14.3	12.6	13.5	14.6	12.2	10.7	12.4
**White blood cell (10**^**3**^**/μl)**	6.80	7.14	6.81	6.94	7.62	5.62	5.64	7.07	5.97	9.84
**Red blood cell (10**^**6**^**/μl)**	3.94	5.14	6.21	5.11	4.21	4.38	5.01	4.31	3.34	4.18
**Platelets (10**^**3**^**/μl)**	451	174	289	384	542	184	290	190	312	327
**Lymphocyte (%)**	24.0	16.8 **(low)**	29.7	30.1	25.9	24.2 **(low)**	20.7	16.5	19.9	17.4
**Monocyte (%)**	5.6	7.4	6.6	8.1 **(high)**	10.0 **(high)**	11.4 **(high)**	12.4 **(high)**	5.0	11.6 **(high)**	5.5
**Neutrophil (%)**	67.5	75.7 **(high)**	56.1	57.6	61.6	63.8	66.4	77.9 **(high)**	63.3	76.6 **(high)**

**Table 2:** Demographics and laboratory examinations data

Variables	Frequency	Percentage (%)
**Age** (mean ± SD)	49.60 ± 20.58 years	
**Gender**		
Male	5	50
Female	5	50
**Comorbidity**		
Yes	5	50
No	5	50
**Vitamin D Status**		
Deficiency	9	90
Insufficiency	1	10
**Lymphocyte**		
Low	2	20
Normal	8	80
High	0	0
**Monocyte**		
Low	0	0
Normal	5	50
High	5	50
**Neutrophil**		
Low	0	0
Normal	7	70
High	3	30

Table 3 shows vitamin D levels based on demographics and laboratory examinations data. The research data were analyzed by using the Fisher’s exact test, resulting that there were no significant results on all variables where *p* > 0.05. These results indicate that all the variables in Table 3 do not significantly affect the occurrence of low vitamin D levels in COVID-19 patients.

**Table 3:** Vitamin D status based on demographics and laboratory examinations data

Variables	Vitamin D		*p* value (Fisher’s exact test)
	Deficiency (%)	Insufficiency (%)	
**Gender**			
Male	40	10	1.00
Female	50	0	
**Comorbidity**			
Yes	50	0	1.00
No	40	10	
**Lymphocyte**			
Low	20	0	1.00
Normal	70	10	
**Monocyte**			
Normal	40	10	1.00
High	50	0	
**Neutrophil**			
Normal	60	10	1.00
High	30	0	

## Discussion

Here, we report our findings in 10 patients with confirmed COVID-19 infection and hospitalized in Bethesda Hospital, Yogyakarta, Indonesia. From the total patients, 50% (5 patients) were males and 50% (5 patients) were females with an average age of 49.6 years. The incubation period for COVID-19 in this case is 2–14 days. Our study showed that the elderly and females tend to have lower vitamin D levels. This is relevant to the facts on vitamin D and COVID-19 in which the 25-hydroxyvitamin D [25(OH)D] concentrations tend to decrease with the age [12]. Furthermore, COVID-19 case fatality rates (CFRs) increase as the age increases [13].

In this case, we report various symptoms of COVID patients such as fatigue, fever, dry cough, headache, non-specific headache, diarrhea, and those who also had no symptoms. All patients in this case had mild to moderate severity. This result is similar with other studies. COVID-19 has a clinical presentation similar to SARS and MERS. The most common symptoms of COVID-19 are fatigue, fever, and breathing disorder, including cough, sore throat, and shortness of breath. Symptoms of intestinal disorders are rarely reported in patients with COVID-19, although diarrhea occurs in about 20–25% of patients with SARS and MERS [14–16].

We found that COVID-19 patients in this case had several comorbidities which are chronic diseases such as hypertension, diabetes, COPD, and post stroke. Some studies reported that people with chronic disease comorbidities have lower 25(OH)D than healthy people [17]. Higher concentrations of 25-hydroxyvitamin D [25(OH)D] have the important benefits and may reduce the risk of many chronic diseases including cancer, hypertension, cardiovascular disease, diabetes mellitus, and chronic respiratory infections [17].

In this case series, we evaluated vitamin D status in 10 patients with COVID-19. The examination of vitamin D levels was carried out by blood tests in the laboratory. From 10 patients, we found that 90% (9 patients) had vitamin D deficiency and 10% (1 patient) had vitamin D insufficiency. Some of the patients had very severe deficiency. The distribution of vitamin D levels in COVID-19 patients in Indonesia is not yet widely available, due to the lack of studies on vitamin D in COVID-19 patients. We can only explain in a study in Indonesia that is currently ongoing, where the prevalence of vitamin D deficiency in COVID-9 patients was 23% [6]. Similar to our findings, previous studies reported COVID-19 patients among ICU subjects in which 11 (84.6%) of them had vitamin D insufficiency vs. 4 (57.1%) of floor subjects. Surprisingly, 100% of ICU patients who are less than 75 years old had vitamin D insufficiency (*n* = 11). Among these, 64.6% (*n* = 7) had very low 25(OH)D levels < 20 ng/mL and three had 25(OH)D levels < 10 ng/mL [18].

A recent study from Martineau et al. performed a meta-analysis from 25 randomized controlled trials (10,933 participants). There was a statistically significant reduction found from vitamin D supplementation in the risk of having acute respiratory infection. In subgroup analysis, they found a protective effect in daily or weekly supplementation but not in bolus doses. There was a strong protective effect in those with 25(OH)D levels < 10 ng/mL and there was no significant effect in those with serum 25(OH)D > 10 ng/mL. There was an inverse relationship between serum vitamin D levels and risk of acute respiratory tract infection [10]. It means vitamin D levels deficiency may contribute to increased risk of respiratory infection including COVID-19 [19].

Previous report have speculated that people with low serum vitamin D might be at higher risk of infection with COVID-19 or will be worsened when being infected [20]. There was an overlap between groups at high risk of vitamin D deficiency and groups at high risk of severe COVID-19. Examples include people with chronic disease and the elderly. Our studies showed that some of the COVID-19 cases were elderly and had a chronic disease.

In Table 3, we analyzed all the variables with the Fisher’s exact test to find out whether vitamin D is related to demographics and laboratory examinations data. From the results of the analysis, we found that all the variables in Table 3 did not cause significant effects in the occurrence of low vitamin D levels in COVID-19 patients. This result can occur because of the small amount of the data. A previous study in Turkey found that vitamin D deficiency was associated with gender and age. In that study, they found as many as 83.8% of women and 18.2% of men have vitamin D deficiency (< 10 ng/mL). Vitamin D insufficiency (10–30 ng/mL) was found in 69.6% of women and 30.4% of men. The main cause of vitamin D deficiency can be associated with a lack of sun exposure [21]. Most studies have found a higher prevalence of vitamin D deficiency among older people [21], perhaps due to the lower skin capacity in older people to produce vitamin D after sun exposure or lack of vitamin D intake in elderly [22], although some studies reported a higher prevalence of vitamin D deficiency in young people [23].

Vitamin D deficiency has been found to contribute to acute respiratory distress syndrome, a major cause of death associated with COVID-19 [24]. Vitamin D plays a role in strengthening the body’s immunity by inducing monocyte differentiation and inhibiting lymphocyte proliferation [25]. A recent randomized meta-analysis of controlled trials concluded that there was a decrease in the total mortality rate in the use of vitamin D supplements [26].

The seasonality of many viral infections, one of them is a respiratory viral infection, is associated with a low concentration of 25(OH)D; thus, UVB doses are low because of winters in temperate climates and rainy seasons related in tropical climates [27]. Our study showed that all patients had the deficient and insufficient status of vitamin D. The surprising fact is that although Indonesia is a tropical country and located in a geographic region which is very rich of sunlight, it can be seen with low vitamin D levels, and the sun cannot be used sufficiently.

Vitamin D has possible beneficial effects on the immune system, especially in COVID-19 patient. For example, vitamin D will increase the production of various peptides by the innate immune system, which has anti-viral, anti-fungal, and anti-microbial activity (e.g., cathelicidin and defensins) [28]. Vitamin D has been proven to not only reduce the production of proinflammatory Th1 cytokines but also to increase the expression of anti-inflammatory cytokines by macrophages. This may be worth bearing in mind that the proinflammatory cytokine environment was observed in patients infected with COVID-19 and how the “cytokine storm” leads to acute respiratory distress syndrome [29].

There are many health benefits of vitamin D as reviewed in a number of reviews. The major cause of vitamin D deficiency globally is an underappreciation of sunlight’s role in providing humans with their vitamin D_3_ requirement. It is estimated that exposure to UV B rays for 1 minimal erythemal dose (MED) is equivalent to ingesting between 10,000 and 25,000 IU of vitamin D. The associations regarding increased risk of developing deadly cancer, infectious diseases, autoimmune disease, cardiovascular disease with living at higher latitudes, and being prone to vitamin D deficiency should remind all health care professionals of the importance of vitamin D for overall health and well-being [30].

A randomized controlled trial investigated monthly supplementation with 100,000 IU vitamin D_3_; there is no risk of kidney stones or did not affect the incidence of hypercalcemia [31]. We also found a case report in USA about the adverse effect s in high-dose supplementation of vitamin D. From the case report, there was a significant manufacture and labeling error, patients had been consuming more than 1000 times the recommended daily dose of vitamin D_3_. But vitamin D intoxication can be managed effectively and usually does not appear to cause long-term sequelae. Hypercalcemia resolves months before normalization of serum 25(OH)D levels [32].

We did find several studies that were registered but have not yet been reported. One trial was tested whether a single oral dose of 25,000 IU (625 μg) of vitamin D would improve mortality in patients who were infected with SARS-CoV-2 but did not have severe symptoms, compared to the usual care [33]. Another randomized controlled trial (RCT) compared single doses of vitamin D_3_, 50,000 IU to 200,000 IU (1250 vs. 5000 μg) in people with COVID-19 pneumonia who are over 75 years old, or over 70 years old, with low oxygen saturations; the primary outcome measure is mortality at 14 days [34].

Review and meta-analysis of RCTs showed vitamin D supplementation had a greater protective effects of vitamin D supplementation taken over daily or weekly to people with the lowest vitamin D levels: the risk of having at least one ARI was reduced from 60 to 32%. Taking vitamin D supplements was found to be safe [35].

We found a current trial of vitamin D in COVID-19 that has reported results. A recent study conducted using a parallel pilot randomized open label, double-masked clinical trial described the treatment outcomes of 50 patients treated with calcifediol and 26 patients who were not treated with calcifediol. From the 50 people who were given calcifediol (patients in the calcifediol treatment group continued with oral calcifediol 0.266 mg on day 3 and 7, and then weekly until discharge or ICU admission), it was found that one patient required admission to the ICU (2%), whereas of the 26 patients who were not treated with calcifediol, and 13 patients were treated with calcifediol (50%), Fisher’s exact test *p* value < 0.001. The end result of the treatment of patients treated with calcifediol, none died, and all were discharged without complications. Meanwhile, 13 patients who were not treated with calcifediol, who were not admitted to the ICU, were discharged. Of the 13 patients admitted to the ICU, two died and the remaining 11 were discharged. Univariate estimated chance ratio for ICU in patients on calcifediol treatment versus without calcifediol treatment was 0.02 (95% CI 0.002–0.17). Multivariate estimated chance ratio for ICU in patients on calcifediol treatment vs. without calcifediol treatment ICU (adjusted for hypertension and T2DM) was 0.03 (95% CI 0.003–0.25) [36].

A recent pilot study has shown that there is a significant effect on administering high doses of calcifediol or 25-hydroxyvitamin D in terms of reducing the need for ICU care in patients requiring hospitalization due to COVID-19. But to show a definite answer postulating that the endocrine system of vitamin D well modulates the host response to severe acute respiratory syndrome coronavirus 2 (SARS-CoV-2), larger trials with appropriate cohorts need to be conducted [36].

We would need evidence from well-masked randomized controlled trials (RCTs) to determine if there are effects of vitamin D_3_ supplements for treating or preventing COVID-19 infection. But actually, it is difficult to conduct vitamin D RCTs since there are many sources of vitamin D [37].

The evidence from observational studies is sufficient to recommend vitamin D supplementation now, since in addition to the observational study evidence. There are many health benefits of vitamin D and very few adverse effects, even at a high dose [30–32]. Therefore, taking vitamin D would very likely reduce risk of COVID-19 and would have other health benefits.

People at risk of vitamin D deficiency should in any case take supplements in line with current guidance. In our case series, we treated all our patients with 2000 IU oral supplementation. As clinicians, we should continue to treat people with vitamin D deficiency but not because of any possible association with a respiratory infection.

## Conclusions

The prevalence of vitamin D deficiency in this study was 90% and only 1 patient (10%) had vitamin D insufficiency. There are many health benefits of vitamin D and very few adverse effects. A hypothetical review showed that vitamin D supplementation may be beneficial for COVID-19. Another randomized controlled trials need to determine and evaluate this recommendation in preventing or treating COVID-19. Clinicians should continue to treat people with vitamin D deficiency especially in managing COVID-19 patients.

## Data Availability

Study’s data can be accessed from RTP after permission and approval from the Bethesda Hospital Yogyakarta, Indonesia.

## Abbreviations

COVID: Coronavirus disease
SARS: Severe acute respiratory syndrome
MERS: Middle East respiratory syndrome
CFR: Case fatality rate
RCT: Randomized controlled trial
